# Supplementary material for: Interpretable, physics-informed learning reveals sulfur adsorption and poisoning mechanisms in 13-atom icosahedra nanoclusters
Source: Sci Rep. 2026 May 4;16:14174. doi: 10.1038/s41598-026-50998-x (PMC13139602; doi:10.1038/s41598-026-50998-x)
Supplement: Supplementary file 1 — Supplementary Information. [file 41598_2026_50998_MOESM1_ESM.pdf]

Supporting Information: Interpretable, Physics-Informed Learning Reveals Sulfur Adsorption and Poisoning Mechanisms in 13-Atom Icosahedra Nanoclusters

Raiane Ferreira Monteiro,[†] João Marcos T. Palheta,[†] Tulio Gnoatto Grison,[†] Octávio Rodrigues Filho,[†] Renato Luis Tame Parreira,[‡] Diego Guedes-Sobrinho,[¶] Celso R. C. Rêgo,[*,§] Alexandre Cavalheiro Dias,[∥] Krys Elly de Araújo Batista,[⊥] and Maurício Jeomar Piotrowski[†]

†*Department of Physics, Federal University of Pelotas, PO Box* 354*, Pelotas, RS,* 96010-900*, Brazil*

‡*Núcleo de Pesquisas em Ciências Exatas e Tecnológicas, Universidade de Franca, Franca, SP, Brazil*

¶*Chemistry Department, Federal University of Paraná, Curitiba, PR, 81531-980, Brazil*

§*Karlsruhe Institute of Technology (KIT), Institute of Nanotechnology, Eggenstein-Leopoldshafen, Germany*

∥*Institute of Physics and International Center of Physics, University of Brasília, Brasília* 70919-970*, DF, Brazil*

⊥*Federal Institute of Amazonas, Manaus, AM,* 69086-475*, Brazil*

E-mail: celso.rego@kit.edu

Contents

I Convergence Tests **S-3**

II Lowest energy TM_{13} and S/TM_{13} **S-4**

A TM_{13} Properties S-6

B S/TM_{13} Properties S-7

III AIMD – Thermalization **S-8**

IV Total Magnetic Moment Values **S-9**

V Machine-Learning Descriptor and Cluster Audit **S-10**

I Convergence Tests

Table S1: Convergence tests for Hg_{13} with regard to the box size (*Box Size*): the relative total energy (ΔE_{tot}), average bond length (d_{av}), effective coordination number (ECN), and total magnetic moment (m_T).

Box Size (Å)	ΔE_{tot} (eV)	ECN	d_{av} (Å)	m_T (μ_B)
12	0.1910	6.3719	2.9493	6.0000
14	0.0088	6.3722	2.9461	6.0000
16	0.0062	6.3940	2.9457	6.0000
18	0.0002	6.3914	2.9454	6.0000
20	0.0001	6.3913	2.9455	6.0000
22	0.0000	6.3914	2.9455	6.0000

Table S2: Convergence tests for Hg_{13} with regard to the cutoff energy (*ENCUT*): the relative total energy (ΔE_{tot}), average bond length (d_{av}), effective coordination number (ECN), and total magnetic moment (m_T).

ENCUT (eV)	ΔE_{tot} (eV)	ECN	d_{av} (Å)	m_T (μ_B)
70.7410	4.1910	6.3709	2.9552	4.0000
141.4820	0.3288	6.3725	2.9472	2.0000
282.9640	0.0962	6.3941	2.9459	6.0000
424.4460	0.0023	6.3913	2.9455	6.0000
495.1870	0.0011	6.3914	2.9454	6.0000
565.9280	0.0000	6.3914	2.9455	6.0000

Table S3: Convergence tests for Hg_{13} with regard to the energy criterion (electronic convergence, *EDIFF*): the relative total energy (ΔE_{tot}), average bond length (d_{av}), effective coordination number (ECN), and total magnetic moment (m_T).

EDIFF (eV)	ΔE_{tot} (eV)	ECN	d_{av} (Å)	m_T (μ_B)
10^{-2}	0.1910	6.3799	2.9498	6.0000
10^{-3}	0.0097	6.3865	2.9459	6.0000
10^{-4}	0.0034	6.3913	2.9454	6.0000
10^{-5}	0.0002	6.3914	2.9455	6.0000
10^{-6}	0.0001	6.3914	2.9455	6.0000
10^{-7}	0.0000	6.3914	2.9455	6.0000

Table S4: Convergence tests for Hg_{13} with regard to the force criterion (ionic convergence, *EDIFFG*): the relative total energy (ΔE_{tot}), average bond length (d_{av}), effective coordination number (ECN), and total magnetic moment (m_T).

EDIFF (eV / Å)	ΔE_{tot} (eV)	ECN	d_{av} (Å)	m_T (μ_B)
0.100	0.1787	6.3701	2.9503	6.0000
0.050	0.0113	6.3987	2.9469	6.0000
0.025	0.0024	6.3912	2.9456	6.0000
0.015	0.0002	6.3913	2.9455	6.0000
0.010	0.0002	6.3913	2.9455	6.0000
0.005	0.0000	6.3914	2.9454	6.0000

II Lowest energy TM_{13} and S/TM_{13}

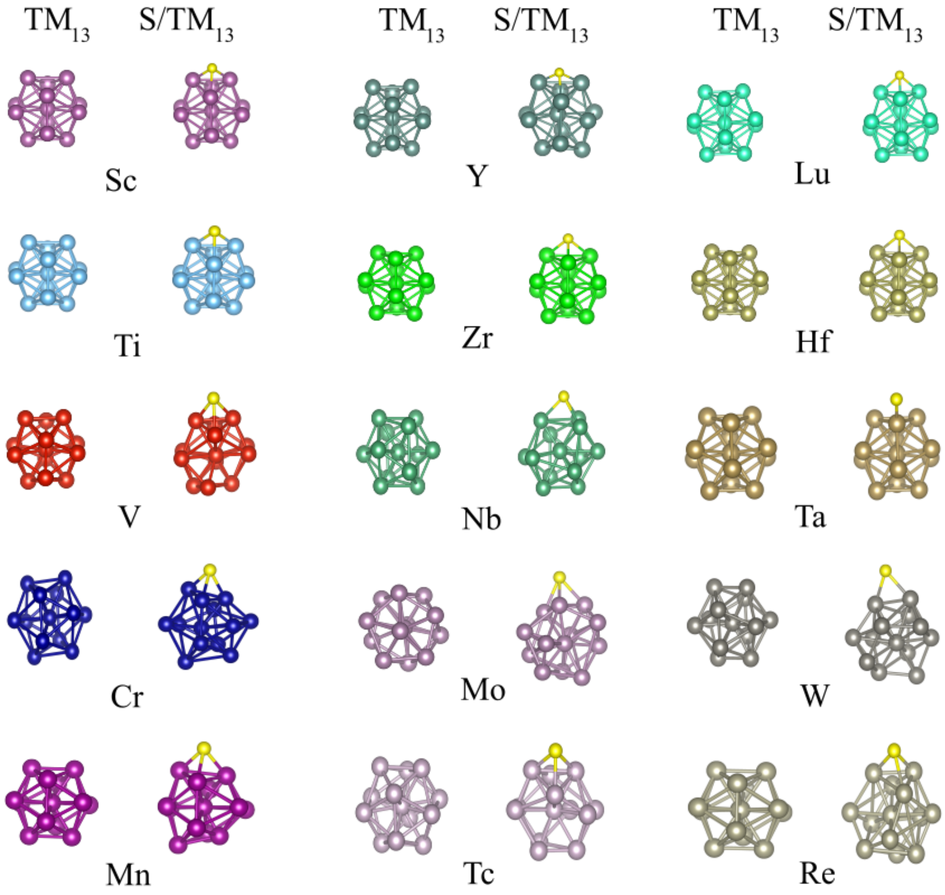

Figure S1 (first part): Lowest energy TM_{13} and S/TM_{13} systems.

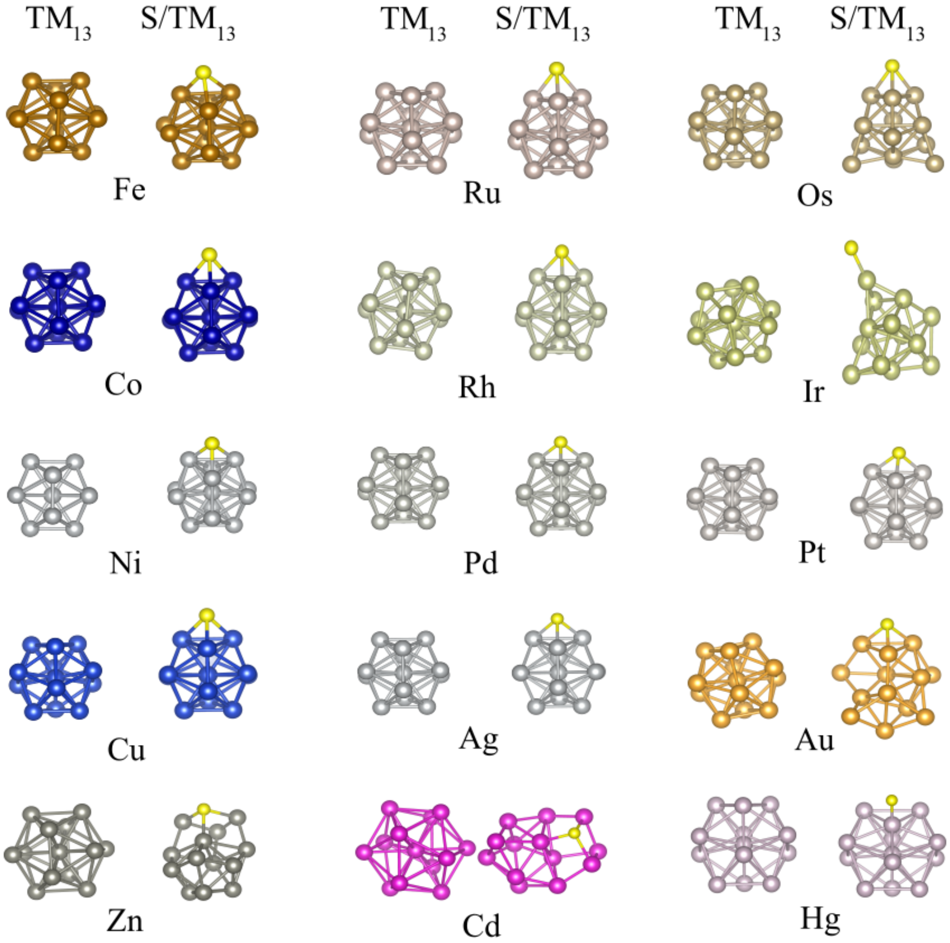

Figure S1 (second part): Lowest energy TM_{13} and S/TM_{13} systems.

A TM$_{13}$ Properties

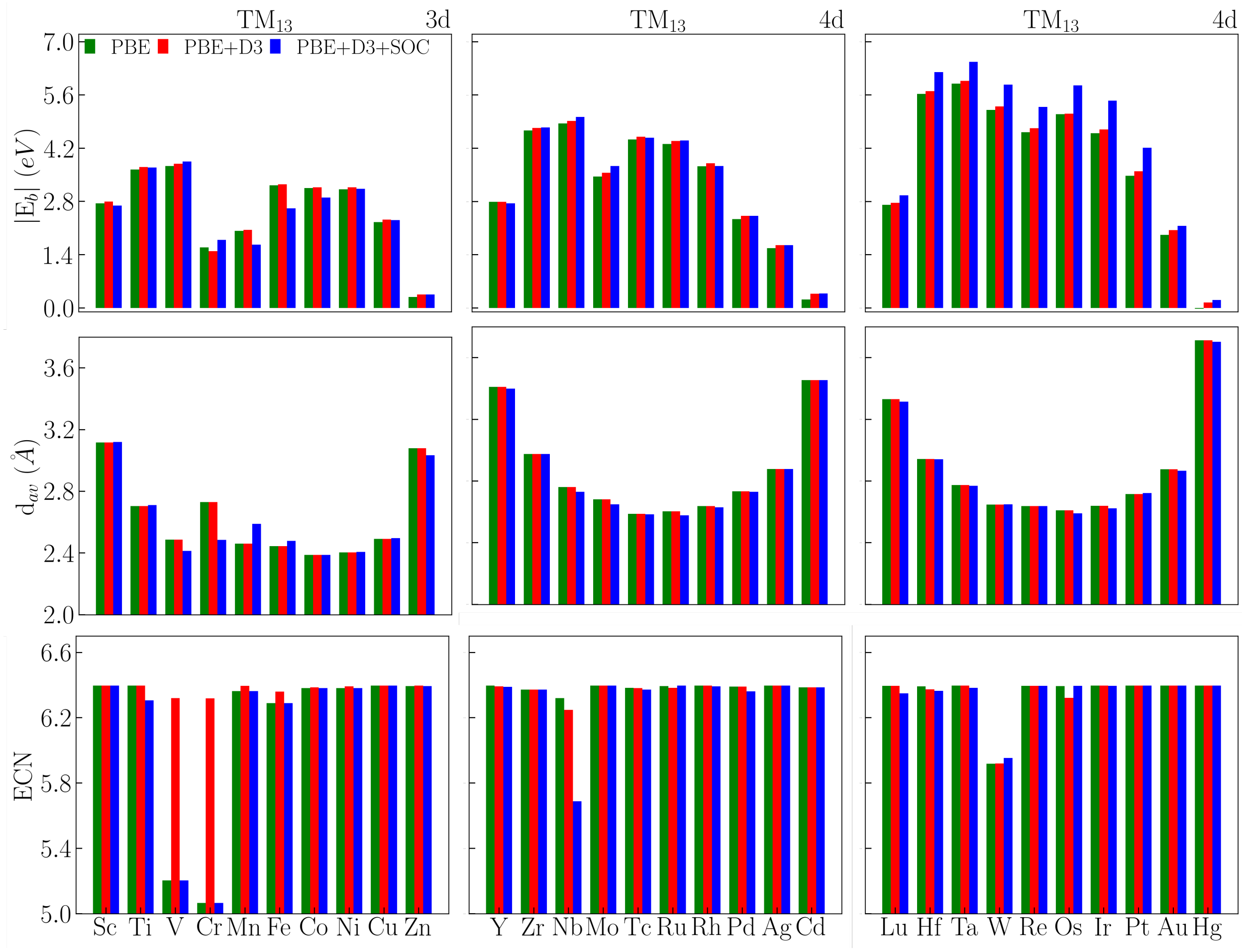

Figure S2: TM$_{13}$ properties: Magnitude of binding energy per atom ($|E_b|$), average bond length (d_{av}), and the effective coordination number (ECN) as a function of atomic number. Considering the PBE, PBE+D3, and PBE+D3+SOC calculation protocols.

B S/TM_{13} Properties

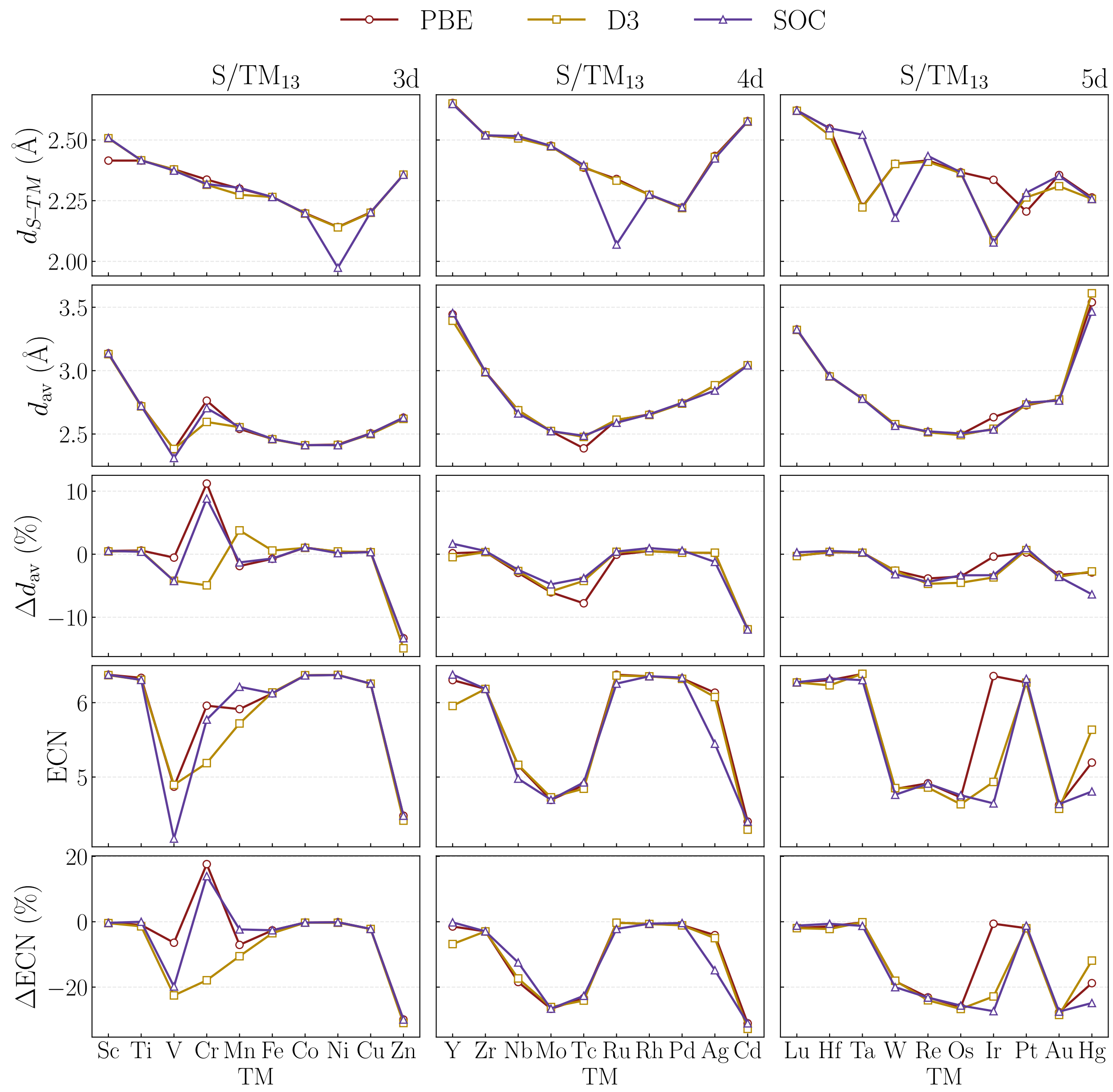

Figure S3: S/TM_{13} properties: Average distance between S and TM atoms (d_{S-TM}), average bond length (d_{av}), the relative variation for d_{av}, effective coordination number (ECN), and the relative variation for ECN as a function of atomic number. Considering the PBE, D3, and SOC calculation protocols.

III AIMD – Thermalization

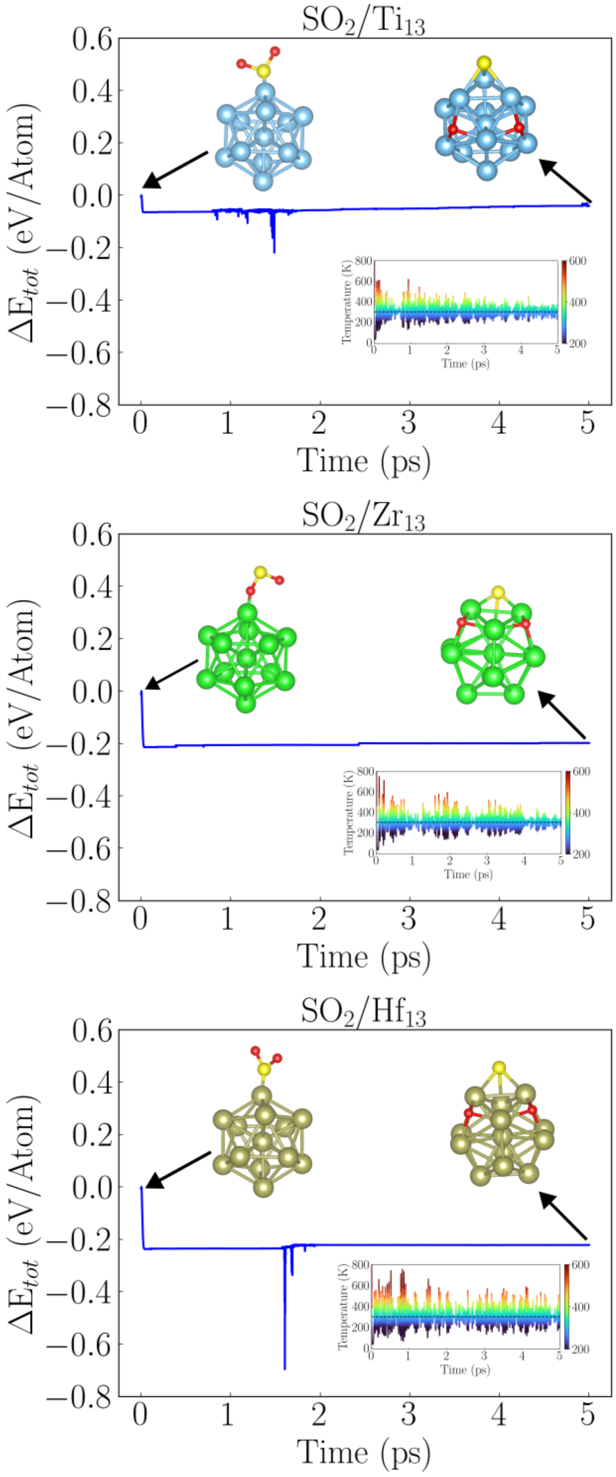

Figure S4: The *Ab Initio* Molecular Dynamics (AIMD), considering thermalization protocol, for SO_2/Ti_{13}, SO_2/Zr_{13}, and SO_2/Hf_{13} systems.

IV Total Magnetic Moment Values

Table S5: Total magnetic moment values, m_T (μ_B) for TM_{13} and S/TM_{13} systems, for all TM elements.

System	TM_{13}	S/TM_{13}	System	TM_{13}	S/TM_{13}	System	TM_{13}	S/TM_{13}
Sc_{13}	19	17	Y_{13}	7	1	Lu_{13}	13	11
Ti_{13}	6	6	Zr_{13}	6	2	Hf_{13}	6	4
V_{13}	7	3	Nb_{13}	3	1	Ta_{13}	7	5
Cr_{13}	50	32	Mo_{13}	20	2	W_{13}	4	4
Mn_{13}	33	29	Tc_{13}	13	1	Re_{13}	13	1
Fe_{13}	34	40	Ru_{13}	18	18	Os_{13}	16	2
Co_{13}	21	27	Rh_{13}	17	17	Ir_{13}	15	7
Ni_{13}	8	8	Pd_{13}	8	4	Pt_{13}	2	2
Cu_{13}	5	1	Ag_{13}	5	1	Au_{13}	5	1
Zn_{13}	0	0	Cd_{13}	0	0	Hg_{13}	0	0

V Machine-Learning Descriptor and Cluster Audit

Tables S6a and S6b shows when the a descriptor is meaningful for both pristine and adsorbed systems (for example d_{av}, ECN, ν_{min}, ν_{max}, E_{ZPE}, ε_d, and E_{gap}), the value corresponding to the specific representation under analysis (TM_{13} or S/TM_{13}) was used. In the supervised regression task, the inputs are restricted to pristine-cluster descriptors, whereas the target is E_{ads} for atomic sulfur adsorption.

Abbreviations: U = unsupervised analysis; I = supervised input; T = supervised target. For descriptors meaningful in both pristine and adsorbed systems, the value corresponding to the analyzed representation was used. In the supervised regression task, only pristine-cluster descriptors were used as inputs, whereas E_{ads} was the target.

Table S6a: Energetic and structural descriptors used in the machine-learning analyses.

Descriptor	Meaning	Units	System	ML role
$E_b^{TM_{13}}$	Per-atom binding energy of the pristine nanocluster; descriptor of intrinsic stability.	eV/atom	TM_{13}	U, I
$E_b^{S/TM_{13}}$	Per-atom binding energy of the sulfur-adsorbed nanocluster.	eV/atom	S/TM_{13}	U
E_{ads}	Atomic-sulfur adsorption energy; negative values indicate exothermic adsorption.	eV	S/TM_{13}	U, T
ΔE_{int}	Frozen-fragment interaction energy between sulfur and the nanocluster.	eV	S/TM_{13}	U
$\Delta E_{dis}^{TM_{13}}$	Per-atom distortion penalty of the nanocluster upon sulfur adsorption.	eV/atom	S/TM_{13}	U
d_{av}	Average TM–TM bond length; tracks the overall cluster size/bonding scale.	Å	Both	U, I
ECN	Effective coordination number; measures local packing/coordination.	–	Both	U, I
Δd_{av}	Relative change in average bond length after adsorption.	%	S/TM_{13}	U
ΔECN	Relative change in effective coordination number after adsorption.	%	S/TM_{13}	U
d_{TM-S}	Minimum metal–sulfur distance in the optimized adsorbed geometry.	Å	S/TM_{13}	U

Table S7 shows that the supervised evaluation is reported using grouped cross-validation rather than a single train/test split because the data set is small and chemically correlated across the transition-metal series. PCA was used only to visualize the organization of the standardized

Table S6b: Vibrational and electronic descriptors used in the machine-learning analyses.

Descriptor	Meaning	Units	System	ML role
ν_{min}	Lowest non-imaginary vibrational frequency; sensitive to soft modes and flexibility.	cm^{-1}	Both	U, I
ν_{max}	Highest vibrational frequency; sensitive to local bond stiffening.	cm^{-1}	Both	U, I
E_{ZPE}	Zero-point energy obtained from the full vibrational spectrum.	eV	Both	U, I
ε_d	Center of gravity of the occupied *d* states; descriptor of metal–adsorbate hybridization propensity.	eV	Both	U, I
E_{gap}	HOMO–LUMO gap; proxy for electronic hardness and reactivity.	eV	Both	U, I
Q^{Bader} / charge transfer	Bader-charge-based descriptor of charge redistribution; for adsorbed systems it tracks net electron transfer toward sulfur.	*e*	Both	U, I

Abbreviations: U = unsupervised analysis; I = supervised input.

descriptor space; the *k*-means classification was performed in the full standardized feature space.

Table S7: Summary of the machine-learning workflow used in this work. This table explicitly separates the unsupervised organization of descriptor space from the supervised regression and LOFO analyses.

Stage	Data representation	Preprocessing / validation	Model / analysis	Main output
Unsupervised organization of pristine clusters	30 pristine TM_{13} systems described by energetic, structural, vibrational, and electronic descriptors	Numerical descriptors standardized with `StandardScaler`	k-means clustering; PCA used only for low-dimensional visualization	Chemically similar groups of pristine nanoclusters in descriptor space
Unsupervised organization of adsorbed clusters	30 optimized S/TM_{13} systems described by adsorption, structural-response, vibrational, and electronic descriptors	Numerical descriptors standardized with `StandardScaler`	k-means clustering; PCA used only for low-dimensional visualization	Grouping of adsorption-response patterns; identification of persistent chemically similar families across pristine and adsorbed spaces
Supervised regression of atomic-sulfur adsorption energetics	Pristine TM_{13} descriptors used as inputs; atomic-sulfur adsorption energy E_{ads} used as target	Descriptors standardized; model assessment performed with group cross-validation instead of a single random split	ElasticNetCV, RidgeCV, and Explainable Boosting Machine (EBM)	Out-of-sample prediction of sulfur adsorption trends from clean-cluster properties
LOFO robustness analysis	Same supervised setup as above, but removing one pristine descriptor at a time	Group cross-validation repeated after deleting each feature	LOFO metrics: ΔR^2, ΔMAE, and ΔRMSE	Ranking of descriptors by robust utility across model classes; identification of redundant or noise-prone features
